# ﻿Unveiling fungi associated with *Castanopsis* woody litter in Yunnan Province, China: Insights into Pleosporales (Dothideomycetes) species

**DOI:** 10.3897/mycokeys.108.127560

**Published:** 2024-08-22

**Authors:** Guang-Cong Ren, Saowaluck Tibpromma, Kai-Xuan Dong, Chen-Xi Gao, Chao-Shan Zhang, Samantha C. Karunarathna, Abdallah M. Elgorban, Heng Gui

**Affiliations:** 1 School of Pharmacy, Guiyang Healthcare Vocational University, Guiyang 550081, China; 2 Guizhou Provincial Engineering Research Center of Medical Resourceful Healthcare Products, Guiyang Healthcare Vocational University, Guiyang 550081, China; 3 Center for Yunnan Plateau Biological Resources Protection and Utilization College of Biological Resource and Food Engineering, Qujing Normal University, Qujing, Yunnan 655011, China; 4 Center of Excellence in Biotechnology Research (CEBR), King Saud University, Riyadh, Saudi Arabia; 5 Center for Mountain Futures(CMF), Kunming Institute of Botany, Chinese Academy of Sciences, Kunming 650201, China; 6 Department of Economic Plants and Biotechnology, Yunnan Key Laboratory for Wild Plant Resources, Kunming Institute of Botany, Chinese Academy of Sciences, Kunming 650201, China

**Keywords:** Hyphomycetes, new species, phylogeny, sexual morph, taxonomy

## Abstract

During a survey of the diversity of lignicolous fungi in Yunnan Province, China, we collected and identified five microfungi species from dead woody litters of *Castanopsis* trees in terrestrial habitats. Through both morphological comparisons and phylogenetic analyses of multi-gene sequences, we identified two taxa as new species and three collections as new host records within Pleosporales. *Pseudolophiostomalincangense***sp. nov.** is introduced as a sexual morph in Lophiostomataceae, *Pleopunctumbaoshanense***sp. nov.** is introduced as a hyphomycetous fungi in Phaeoseptaceae, and *Paraphomaaquatica* as a first report of sexual morph in *Paraphoma*. In addition, *Occultibambusakunmingensis* and *Pleopunctummegalosporum* were isolated for the first time from the dead twigs of *Castanopsisdelavayi* and *C.calathiformis*, respectively. Comprehensive morphological descriptions, illustrations, and phylogenetic analysis results are provided for the above-mentioned species.

## ﻿Introduction

*Castanopsis* is an evergreen tree belonging to Fagaceae and represents one of the largest genera with approximately 134 species, predominantly distributed across tropical and subtropical regions of Asia (Tan et al. 2023). Notably, 58 species are native to China, with 30 being endemic. *Castanopsis* species are valued for their timber and edible nuts, contributing significantly to the economy (Huang et al. 1999; Tang et al. 2005). Fungal diversity associated with *Castanopsis* has been extensively documented across various countries, including China, Korea, India, Indonesia, Japan, Nepal, Papua New Guinea, Thailand, and the United States (Crawford et al. 1987; Tang et al. 2005; Duong et al. 2008; Osono et al. 2020; Jayawardena et al. 2020). The presence of fungi on *Castanopsis* trees has been widely introduced, with approximately 360 records worldwide representing 220 species across 35 different *Castanopsis* species (Duong et al. 2008). Tang et al. (2005) reported 38 fungal taxa during a study on decaying leaves of *Castanopsisfissa* in Hong Kong, China highlighting the rich fungal diversity within this genus. These fungi encompass endophytes and saprobes found on different parts of *Castanopsis* trees, such as ectomycorrhizal, woody branches, fallen trunks, bark, and leaves (Inácio et al. 2005; Gao et al. 2016; Hyde et al. 2016; Ren et al. 2022; Pang et al. 2023).

Yunnan Province, located in southwestern China, boasts significant biological diversity attributed to its complex topography, highly variable climate, and lush vegetation (Feng and Yang 2018). This region covers an extensive area of 394,000 square with approximately 94% comprising mountainous terrain (Asian Development Bank 2012). Over the past decade, there has been a surge in interest in studying microfungi in Yunnan Province, with numerous studies focusing on leaf litter fungi and lignicolous freshwater fungi (Cai et al. 2002; Luo et al. 2018; Hapuarachchi et al. 2019; Dong et al. 2020). Recent discoveries have unveiled several new taxa from Dothideomycetes and Sordariomycetes inhabiting woody litter in terrestrial habitats, such as Diatrypaceae, Didymosphaeriaceae, Hermatomycetaceae, Hysteriaceae, Monoblastiaceae and Phaeoseptaceae (Maharachchikumbura et al. 2021; Mortimer et al. 2021; Ren et al. 2022; Wanasinghe and Mortimer et al. 2022). However, many studies lack proper identification and phylogenetic data, underscoring the need to re-evaluate various species in this region (Mortimer et al. 2021).

Pleosporales was established by Barr (1987) and is recognized as the largest order within the class Dothideomycetes, constituting a quarter of all species (Wijayawardene et al. 2022). This order has a remarkable diversity, comprising 91 families and 614 genera (Wijayawardene et al. 2022). Taxonomically, pleosporalean taxa exhibit versatility in ecological niches, being found as epiphytes, endophytes, parasites, hyperparasites, lichenized organisms, or saprobes across a wide range of habitats worldwide (Wanasinghe et al. 2020; Yang et al. 2023). Studies have highlighted the discovery of numerous new pleosporalean species from freshwater, marine, and terrestrial environments (Brahmanage et al. 2020). Morphologically, the sexual morph of Pleosporales is characterized by perithecial ascomata, typically with a papillate apex, ostiolate, cellular pseudoparaphyses, and bitunicate asci. The asexual morphs encompass both coelomycetes and hyphomycetes (Hongsanan et al. 2020).

The present study aims to describe two novel fungal species and three new host records collected from dead woody litter in Baoshan and Lincang of Yunnan Province, China. This involves morphological illustrations and multi-gene phylogenetic analyses utilizing ML and BI methods to confirm the phylogenetic placement. The study aims to contribute to fungal diversity and ecology in Yunnan Province while providing valuable insights into the taxonomy and phylogenetics of woody litter fungi.

## ﻿Materials and methods

### ﻿Sample collection, observation, and isolation

Decayed woody samples were collected from mixed forest areas in China (Yunnan Province) during the rainy season (July) and brought to the laboratory in separate zip-lock plastic bags. Specimens were examined using a stereomicroscope (Olympus SZ61, Tokyo, Japan). Micro-morphological characteristics were photographed using a Canon EOS 600D (Tokyo, Japan) digital camera mounted on a Nikon ECLIPSE 80i (Tokyo, Japan) compound microscope. All microscopic measurements were taken using the Tarosoft (R) Image Frame Work v.09, and the measurements were reported as minimum–maximum values and average values. Images were processed with Adobe Photoshop CS6 software v.13 (Adobe Systems, San Jose, CA, USA). Single-spore isolation was used to obtain pure cultures, following the methods described by Ren et al. (2022). Herbarium materials were deposited at the Herbarium of Cryptogams Kunming Institute of Botany, Academia Sinica (HKAS), Kunming, China, and living cultures were deposited at the Kunming Institute of Botany Culture Collection (KUNCC), Kunming, China. Faces of fungi (Jayasiri et al. 2015) and Index Fungorum (2024) numbers were obtained for the new taxa.

### ﻿DNA extraction, PCR amplification, and sequencing

Genomic DNA was extracted from the mycelium grown on PDA at 25 °C for four weeks using Biospin Fungus Genomic DNA Extraction Kit (BioFlux®) (Hangzhou, P.R. China). Five gene regions, including internal transcribed spacer region (ITS), large subunit nuclear ribosomal (LSU), small subunit ribosomal RNA (SSU), translation elongation factor 1-alpha gene (*tef*1-α), and RNA polymerase II second largest subunit (*rpb*2) were amplified with primers ITS5/ITS4 (White et al. 1990), LR0R/LR5 (Vilgalys and Hester 1990), NS1/NS4 (White et al. 1990), 983F/2218R (Rehner and Buckley 2005) and fRPB2-5F/fRPB2-7cR (Liu et al. 1999), respectively. The PCR thermal cycle programs for SSU, LSU, ITS, *tef*1-α, and *rpb*2 were set as described in Wanasinghe et al. (2021). The quality of PCR products was checked on 1% agarose gel electrophoresis stained with ethidium bromide. The PCR products were sent for sequencing at Qingke Company, Kunming City, Yunnan Province, China. The sequences were deposited in GenBank.

### ﻿Phylogenetic analyses

Sequences exhibiting high similarities (>90%) were identified through BLASTn searches to determine the closest match to the taxa. Representative sequences were individually blasted and the initial results from BLASTn searches show our five taxa belong to Pseudolophiostoma in Lophiostomataceae, *Occultibambusa* in Occultibambusaceae, *Pleopunctum* in Phaeoseptaceae, and *Paraphoma* in Phaeosphaeriaceae. Thus, four different datasets were prepared and analysed in this study based on recent publications (Gomzhina et al. 2020; Phukhamsakda et al. 2020; Magaña-Dueñas et al. 2021; Guarnaccia et al. 2022; Yu et al. 2022; Xu et al. 2023). The sequences were downloaded from GenBank (http://www.ncbi.nlm.nih.gov/) and the accession numbers are listed in Table 1. The newly generated sequences were assembled by BioEdit 7.2.3 (Hall 1999). The individual gene regions were separately aligned in the MAFFT v.7 web server (http://mafft.cbrc.jp/alignment/server/) (Katoh et al. 2019). The alignments of each gene were improved by manually deleting the ambiguous regions plus gaps and combined using BioEdit 7.2.3. The final alignments were converted to NEXUS format (.nxs) using Clustal X version 1.81 (Thompson et al. 1997) and processed for Bayesian and maximum parsimony analysis. The FASTA format was changed into PHY format via the Alignment Transformation Environment (ALTER) online program (http://www.sing-group.org/ALTER/) and used for maximum likelihood (ML) analysis.

**Table 1.** Names, strain numbers, and corresponding GenBank accession numbers of the taxa used in the phylogenetic analysis.

Taxon name	Strain number	GenBank accession numbers					
		SSU	ITS	LSU	*tef*1-α	*rpb*2	*tub*2
*Alpestrisphaeriajonesii*	GAAZ 54-1	KX687755	KX687757	KX687753	KX687759	NA	NA
*Alpestrisphaeriajonesii*	GAAZ 54-2	KX687756	KX687758	KX687754	KX687760	NA	NA
*Alpestrisphaeriaterricola*	SC-12^T^	JX985749	JN662930	JX985750	NA	NA	NA
*Angustimassarinaacerina*	MFLUCC 14-0505^T^	NG_063573	KP888637	NR_138406	KR075168	NA	NA
*Angustimassarinapopuli*	MFLUCC 13-0034^T^	NG_061204	KP888642	KP899137	KR075164	NA	NA
*Biappendiculisporajaponica*	KT 573^T^	AB618686	LC001728	AB619005	LC001744	NA	NA
*Biappendiculisporajaponica*	KT 794	AB618688	LC001730	AB619007	LC001746	NA	NA
*Biappendiculisporajaponica*	KT 686	AB618687	LC001729	AB619006	LC001745	NA	NA
*Brunneofusisporaclematidis*	MFLUCC 17-2070	MT226685	MT310615	MT214570	MT394692	MT394629	NA
*Brunneofusisporainclinatiostiola*	CGMCC 3.20403	MZ964884	MZ964866	MZ964875	OK061075	OK061069	NA
*Brunneofusisporasinensis*	KUMCC 17-0030	MH393556	MH393558	MH393557	NA	MH395329	NA
*Capulatisporasagittiforme*	KT 1934^T^	AB618693	AB369268	AB369267	LC001756	NA	NA
*Coelodictyosporiummuriforme*	MFLUCC 13-0351^T^	KP899127	KP899136	KP888641	KR075163	NA	NA
*Coelodictyosporiumpseudodictyosporium*	MFLUCC 13-0451^T^	NA	KR025858	KR025862	NA	NA	NA
*Crassiclypeusaquaticus*	KT 970^T^	LC312472	LC312501	LC312530	LC312559	LC312588	NA
*Crassiclypeusaquaticus*	KH 104	LC312470	LC312499	LC312528	LC312557	LC312586	NA
*Decaisnellaformosa*	BCC 25616	GQ925833	GQ925846	NA	GU479851	NA	NA
*Decaisnellaformosa*	BCC 25617	GQ925834	GQ925847	NA	GU479850	NA	NA
*Desertiserpenticahydei*	SQUCC 15092^T^	MW077163	MW077147	MW077156	MW075773	NA	NA
*Dimorphiopsisbrachystegiae*	CPC 22679^T^	NA	KF777160	KF777213	NA	NA	NA
*Ernakulamiakrabiensis*	MFLUCC 18-0237	MK347880	MK347773	MK347990	NA	NA	NA
*Ernakulamiaxishuangbannaensis*	KUMCC 17-0187	MH260354	MH275080	MH260314	NA	NA	NA
*Flabellascomaaquaticum*	KUMCC 15-0258	MN304832	MN304827	MN274564	MN328898	MN328895	NA
*Flabellascomacycadicola*	KT 2034^T^	LC312473	LC312502	LC312531	LC312560	LC312589	NA
*Flabellascomafusiforme*	MFLUCC 18-1584	NA	MN304830	MN274567	MN328902	NA	NA
*Guttulisporacrataegi*	MFLUCC 14-0993	KP899126	KP899135	KP888640	KR075162	NA	NA
*Guttulisporacrataegi*	MFLUCC 13-0442^T^	KP899125	KP899134	KP888639	KR075161	NA	NA
*Kiskunsagiaubrizsyi*	REF121^T^	MK589351	JN859341	MK589359	MK599325	NA	NA
*Lentistomaaquaticum*	MFLUCC 18-1275	MT864320	MT627697	MN913723	MT954370	NA	NA
*Lentistomabipolare*	KT 3056	LC312484	LC312513	LC312542	LC312571	LC312600	NA
*Lentistomabipolare*	CBS 115375	LC312477	LC312506	LC312535	LC312564	LC312593	NA
*Leptopariesmagnoliae*	MFLU 18-1291	ON870915	ON878077	ON870390	NA	NA	NA
*Leptopariespalmarum*	KT 1653^T^	LC312485	LC312514	LC312543	LC312572	LC312601	NA
*Leptosphaeriaheterospora*	AFTOL-ID 1036	NA	GQ203795	AY016369	DQ497609	DQ497615	NA
*Lignosphaeriafusispora*	MFLUCC 11-0377^T^	NA	KP888646	NR_164233	NA	NA	NA
*Lophiohelichrysumhelichrysi*	IT-1296^T^	KT333437	KT333435	KT333436	KT427535	NA	NA
*Lophiopoaceaparamacrostomum*	MFLUCC 11-0463^T^	KP899122	NA	KP888636	NA	NA	NA
*Lophiopoaceawinteri*	KT 740	AB618699	JN942969	AB619017	LC001763	JN993487	NA
*Lophiopoaceawinteri*	KT 764	AB618700	JN942968	AB619018	LC001764	JN993488	NA
*Lophiostomamultiseptatum*	CBS 623.86	GU296163	NA	GU301833	NA	GU371791	NA
*Lophiostomamultiseptatum*	KT 604/JCM17668^T^	AB618684	LC001726	AB619003	LC001742	NA	NA
*Lophiostomasemiliberum*	KT 622	AB618694	JN942966	AB619012	LC001757	JN993483	NA
*Lophiostomasemiliberum*	KT 652	AB618695	JN942967	AB619013	LC001758	JN993485	NA
*Neooccultibambusachiangraiensis*	MFLUCC 12-0559	KU712458	KU712442	KU764699	NA	KU872761	NA
*Neooccultibambusakaiyangensis*	CGMCC 3.20404	MZ964886	MZ964868	MZ964877	OK061077	OK061071	NA
*Neooccultibambusatrachycarpi*	CGMCC 3.20405	MZ964888	MZ964870	MZ964879	OK061079	OK061073	NA
*Neopaucisporarosae-ecae*	MFLUCC 17-0807^T^	MG829139	MG828924	MG829033	MG829217	NA	NA
*Neotrematosphaeriabiappendiculatum*	KTC 975	GU205254	NA	GU205228	NA	NA	NA
*Neotrematosphaeriabiappendiculatum*	KTC 1124^T^	GU205256	NA	GU205227	NA	NA	NA
*Neovaginatisporaclematidis*	MFLUCC 17-2149	MT226676	MT310606	MT214559	MT394738	NA	NA
*Neovaginatisporafuckelii*	MFLUCC 17-1334	MN304833	MN304828	MN274565	MN328899	MN328896	NA
*Neovaginatisporafuckelii*	KT 634	AB618690	LC001732	AB619009	LC001750	NA	NA
*Occultibambusaaquatica*	MFLUCC 11-0006	KX698112	KX698114	KX698110	NA	NA	NA
*Occultibambusabambusae*	MFLUCC 13-0855	KU872116	KU940123	KU863112	KU940170	KU940193	NA
*Occultibambusachiangraiensis*	MFLUCC 16-0380	KX655551	NA	KX655546	KX655566	KX655561	NA
*Occultibambusafusispora*	MFLUCC 11-0127	NA	KU940125	KU863114	KU940172	KU940195	NA
*Occultibambusahongheensis*	KUMCC 21-0020	MZ329029	MZ329037	MZ329033	NA	MZ325467	NA
*Occultibambusajonesii*	GZCC 16-0117	KY628324	NA	KY628322	KY814758	KY814756	NA
*Occultibambusakunmingensis*	HKAS 102151	MT864342	MT627716	MN913733	MT878453	MT954407	NA
***Occultibambusakunmingensis***	**KUNCC 21-0506**	**PP779901**	**PP779906**	**PP779897**	**PP778371**	**NA**	**NA**
*Occultibambusamaolanensis*	GZCC 16-0116	KY628325	NA	KY628323	KY814759	KY814757	NA
*Occultibambusapustula*	MFLUCC 11-0502	KU872118	KU940126	KU863115	NA	NA	NA
*Occultibambusasichuanensis*	CGMCC 3.20938	NA	ON332913	ON332931	ON383989	ON381181	NA
*Occultibambusasichuanensis*	UESTCC 22.0004	NA	ON332914	ON332932	ON383990	ON381182	NA
*Parapaucisporapseudoarmatispora*	KT 2237	LC100018	LC100021	LC100026	LC100030	NA	NA
*Paraphomaaquatica*	FMR 16956^T^	NA	OU612361	OU612360	OU612357	OU612356	OU612355
***Paraphomaaquatica***	**KUNCC 21-0523**	**NA**	**PP779905**	**PP779896**	**PP778370**	**NA**	**NA**
*Paraphomachlamydocopiosa*	UMPc01	NA	KU999072	NA	NA	KU999080	KU999084
*Paraphomachrysanthemicola*	CBS 172.70	NA	KF251165	KF251669	KF252173	KF253123	KF252660
*Paraphomachrysanthemicola*	CBS 522.66^T^	NA	KF251166	KF251670	KF252174	KF253124	KF252661
*Paraphomaconvolvuli*	MF-9.222	NA	MG764055	MG764069	NA	NA	NA
*Paraphomaconvolvuli*	MF-9.265	NA	MG764062	MG764071	MG779467	NA	MG779457
*Paraphomaconvolvuli*	MF-9.298.1	NA	MG764057	MG764074	MG779468	NA	MG779459
*Paraphomaconvolvuli*	MF-9.300.1	NA	MG764064	MG764066	MG779469	NA	MG779460
*Paraphomaconvolvuli*	MF-9.301.1	NA	MG764060	MG764075	MG779470	KF253126	MG779461
*Paraphomadioscoreae*	CPC 11361	NA	KF251169	KF251673	KF252177	KF253127	KF252664
*Paraphomadioscoreae*	CBS 135100	NA	KF251167	KF251671	KF252175	NA	KF252662
*Paraphomafimeti*	CBS 170.70^T^	NA	KF251170	KF251674	KF252178	KF253128	KF252665
*Paraphomafimeti*	CBS 368.91	NA	KF251171	KF251675	KF252179	KF253129	KF252666
*Paraphomagaribaldii*	CBS 148459	NA	OL435708	NA	NA	OL449256	OL449254
*Paraphomagaribaldii*	CBS 148460	NA	OL435709	NA	NA	OL449257	OL449255
*Paraphomaledniceana*	CBS 146533	NA	MT371091	MT371396	MT372655	MT372654	MT372661
*Paraphomamelnikiae*	MF-9.88	NA	MG764063	MG764065	MG779466	NA	MG779456
*Paraphomamelnikiae*	MF-9.95	NA	MG764054	MG764067	MG779462	NA	NA
*Paraphomamelnikiae*	MF-9.182.1	NA	MG764058	MG764068	MG779463	NA	MG779454
*Paraphomamelnikiae*	MF-9.240	NA	MG764061	MG764070	MG779464	NA	MG779453
*Paraphomamelnikiae*	MF-9.296.1	NA	MG764056	NA	MG779465	NA	MG779458
*Paraphomapye*	UMPp04; BRIP 65171	NA	KU999075	NA	NA	NA	KU999087
*Paraphomapye*	UMPp02	NA	KU999073	NA	NA	KU999081	KU999085
*Paraphomaradicina*	CBS 111.79	NA	KF251172	KF251676	KF252180	KF253130	KF252667
*Paraphomaradicina*	CBS 102875^T^	NA	KF251173	KF251677	KF252181	KF253131	KF252668
*Paraphomarhaphiolepidis*	CBS 142524^T^	NA	KY979758	KY979813	KY979851	KY979896	KY979924
*Paraphomasalicis*	CBS 146797	NA	MW883437	MW883829	MW890069	NA	MW890140
*Paraphomavariabilis*	CBS 147695^T^	NA	LR993310	LR993311	LR993313	NA	LR993314
*Paraphomavinacea*	UMPV004	NA	KU176887	KU176891	NA	NA	KU176895
*Paucisporakunmingense*	MFLUCC 17-0932^T^	MF173430	MF173432	MF173428	MF173434	MF173436	NA
*Paucisporaquadrispora*	KT 843^T^	AB618692	LC001734	AB619011	LC001755	NA	NA
*Phaeoseptumaquaticum*	CBS 123113^T^	NA	JN644072	KY940803	NA	NA	NA
*Phaeoseptumcarolshearerianum*	NFCCI-4221^T^	MK307816	MK307813	MK307810	MK309874	MK309877	NA
*Phaeoseptumcarolshearerianum*	NFCCI-4384	MK307818	MK307815	MK307812	MK309876	MK309879	NA
*Phaeoseptumhydei*	MFLUCC 17-0801^T^	MT240624	MT240623	MT240622	MT241506	NA	NA
*Phaeoseptummali*	HKAS122916	ON009082	ON009098	ON009114	ON009257	ON009282	NA
*Phaeoseptummali*	HKAS122917	ON009083	ON009099	ON009115	ON009258	ON009283	NA
*Phaeoseptummali*	MFLUCC 17-2108^T^	NA	MK625197	MK659580	MK647990	NA	NA
*Phaeoseptummanglicola*	NFCCI-4666 T	MK307817	MK307814	MK307811	MK309875	MK309878	NA
*Phaeoseptumterricola*	MFLUCC 10-0102^T^	MH105780	MH105779	MH105778	MH105781	NA	NA
*Platystomumcrataegi*	MFLUCC 14-0925^T^	KT026113	KT026117	KT026109	KT026121	NA	NA
*Platystomumrosae*	MFLUCC 15-0633^T^	KT026115	KT026119	KT026111	NA	NA	NA
*Platystomumsalicicola*	MFLUCC 15-0632^T^	KT026114	KT026118	KT026110	NA	NA	NA
***Pleopunctumbaoshanense***	**KUNCC 21-0494^T^**	**PP779898**	**PP779902**	**PP779893**	**PP778367**	**PP778372**	**NA**
*Pleopunctumclematidis*	MFLUCC 17-2091	NA	MT214573	MT310618	MT394632	MT394693	NA
*Pleopunctumellipsoideum*	MFLUCC 19-0390^T^	MK804514	MK804517	MK804512	MK828510	NA	NA
*Pleopunctumellipsoideum*	MFLUCC 21-0064	NA	OM258687	OM250079	NA	NA	NA
*Pleopunctumguizhouense*	GZCC 23-0595	NA	OR091332	OR098710	NA	NA	NA
*Pleopunctumheveae*	MFLUCC 21-0146	NA	OL782070	OL780491	NA	NA	NA
*Pleopunctummegalosporum*	KUNCC 10785^T^	NA	OQ146985	ON261162	OQ943186	NA	NA
*Pleopunctummegalosporum*	KUNCC 10442	NA	OQ146986	OQ135180	OQ943187	NA	NA
***Pleopunctummegalosporum***	**KUNCC 21-0622**	**PP779899**	**PP779903**	**PP779894**	**PP778368**	**PP778373**	**NA**
*Pleopunctummenglaense*	KUMCC 21-0026^T^	ON009087	ON009103	ON009119	ON009262	ON009287	NA
*Pleopunctummenglaense*	KUMCC 21-0025	ON009086	ON009102	ON009118	ON009261	ON009286	NA
*Pleopunctummulticellularum*	KUNCC 10789^T^	NA	OQ146989	ON261166	OQ943190	NA	NA
*Pleopunctummulticellularum*	KUNCC 10781	NA	OQ146981	ON261158	OQ943189	NA	NA
*Pleopunctummulticellularum*	KUNCC 10778	NA	OQ146978	ON261155	NA	NA	NA
*Pleopunctumpseudoellipsoideum*	MFLUCC 19-0391^T^	NA	MK804518	MK804513	MK828511	NA	NA
*Pleopunctumpseudoellipsoideum*	HKAS122915	ON009085	ON009101	ON009117	ON009260	ON009285	NA
*Pleopunctumrotundatum*	KUNCC 10787^T^	NA	OQ146987	ON261164	OQ943194	NA	NA
*Pleopunctumrotundatum*	KUNCC 10780	NA	OQ146980	ON261157	OQ943193	NA	NA
*Pleopunctumthailandicum*	MFLUCC 21-0039^T^	NA	MZ198896	MZ198894	MZ172461	NA	NA
*Pleopunctumpseudoellipsoideum*	KUMCC 21-0820	ON009084	ON009100	ON009116	ON009259	ON009284	NA
*Pseudocapulatisporaclematidis-subumbellatae*	MFLUCC 17-2063	MT226677	MT310607	MT214560	MT394739	MT394687	NA
*Pseudocapulatisporalongiappendiculatum*	MFLUCC 17-1452^T^	MT214415	MT214368	MT214462	MT235783	NA	NA
*Pseudocapulatisporalongiappendiculatum*	MFLUCC 17-1457	MT214416	MT214369	MT214463	MT235784	MT235821	NA
*Pseudolophiostomachiangraiense*	MFLUCC 17–2076^T^	MT226678	MT310608	MT214561	MT394740	MT394688	NA
*Pseudolophiostomaclematidis*	MFLUCC 17-2081	MT226679	MN393004	MT214562	MT394741	MT394689	NA
*Pseudolophiostomacornisporum*	KH 322^T^	LC312486	LC312515	LC312544	LC312573	LC312602	NA
***Pseudolophiostomalincangense***	**KUNCC 21-0606^T^**	**PP779900**	**PP779904**	**PP779895**	**PP778369**	**PP778374**	**NA**
*Pseudolophiostomamangiferae*	MFLUCC 17-2651^T^	MG931028	MG931031	MG931025	NA	NA	NA
*Pseudolophiostomamangiferae*	MFLUCC 17-2653	MG931029	MG931032	MG931026	NA	NA	NA
*Pseudolophiostomaobtusisporum*	KT 3098	LC312490	LC312519	LC312548	LC312577	LC312606	NA
*Pseudolophiostomaobtusisporum*	KT 2838^T^	LC312489	LC312518	LC312547	LC312576	LC312605	NA
*Pseudolophiostomatropicum*	KH 352	LC312492	LC312521	LC312550	LC312579	LC312608	NA
*Pseudolophiostomatropicum*	KT 3134^T^	LC312493	LC312522	LC312551	LC312580	LC312609	NA
*Pseudolophiostomavitigenum*	HH 26930^T^	AB618697	LC001735	AB619015	LC001761	NA	NA
*Pseudolophiostomavitigenum*	HH 26931	AB618698	LC001736	AB619016	LC001762	NA	NA
*Pseudopaucisporabrunneospora*	KH 227^T^	LC312494	LC312523	LC312552	LC312581	LC312610	NA
*Pseudoplatystomumscabridisporum*	BCC 22835	NA	NA	GQ925844	GU479857	GU479830	NA
*Pseudoplatystomumscabridisporum*	BCC 22836	NA	NA	GQ925845	GU479856	GU479829	NA
*Quintarialignatilis*	CBS 117700	GU296188	NA	GU301865	NA	GU371761	NA
*Quintarialignatilis*	BCC 17444	GU479764	NA	GU479797	GU479859	GU479832	NA
*Seriascomabambusae*	KUMCC 21-0021	MZ329031	MZ329039	MZ329035	MZ325470	MZ325468	NA
*Seriascomadidymosporum*	MFLUCC 11-0179	KU872119	KU940127	KU863116	KU940173	KU940196	NA
*Seriascomayunnanense*	MFLU 19-0690	MN174694	NA	MN174695	MN210324	MN381858	NA
*Setophomaterrestris*	CBS 335.29	NA	KF251246	NA	NA	KF253196	KF252729
*Sigarisporacoronillae*	MFLUCC 14-0941^T^	KT026116	KT026120	KT026112	NA	NA	NA
*Sigarisporajunci*	MFLUCC 14-0938^T^	MG829178	MG828966	MG829078	NA	NA	NA
*Sigarisporaravennicum*	MFLUCC 14-0005^T^	KP698415	KP698413	KP698414	NA	NA	NA
*Sigarisporascrophulariicola*	MFLUCC 17-0689^T^	NA	MG828969	MG829081	NA	NA	NA
*Teichosporarubriostiolata*	TR7	NA	KU601590	KU601590	KU601609	KU601599	NA
*Teichosporatrabicola*	C134	NA	KU601591	KU601591	KU601601	KU601600	NA
*Thyridariamacrostomoides*	GKM 224N	NA	GU385191	NA	GU327777	NA	NA
*Thyridariamacrostomoides*	GKM 1033	NA	GU385190	NA	GU327776	NA	NA
*Thyridariamacrostomoides*	GKM 1159	NA	GU385185	NA	GU327778	NA	NA
*Vaginatisporaappendiculata*	MFLUCC 13-0835^T^	KY264749	NA	KY264745	NA	NA	NA
*Vaginatisporaaquatica*	MFLUCC 11-0083^T^	KJ591575	KJ591577	KJ591576	NA	NA	NA
*Vaginatisporascabrispora*	KT 2443^T^	LC312496	LC312525	LC312554	LC312583	LC312612	NA
*Versicolorisporiumtriseptatum*	JCM 14775	AB524501	AB365596	AB330081	NA	NA	NA
*Versicolorisporiumtriseptatum*	UESTCC 21.0016	OL741381	OL741378	OL741318	NA	NA	NA

The newly generated sequences are indicated in bold. T refers to ex-type strains, and NA refers to “no data in GenBank”.

The maximum likelihood (ML) analysis was performed on the CIPRES Science Gateway v.3.3 (http://www.phylo.org/portal2/; Miller et al. 2010) using RAxML-HPC2 on XSEDE v.8.2.12 (Stamatakis 2014) with parameters adjusted for 1000 bootstrap iterations and the GTRGAMMA substitution model. Bayesian inference was performed in MrBayes v.3.2.7a (Ronquist et al. 2012) using Markov chain Monte-Carlo sampling (BMCMC) to determine posterior probabilities (PPs) (Rannala and Yang 1996). The model of evolution for each gene was estimated using MrModeltest v.2.3 (Nylander et al. 2008) via PAUP v.4.0b10 (Ronquist and Huelsenbeck 2003). Six simultaneous Markov chains were run for 2,000,000 generations, with trees sampled at every 200 generations, until it was stopped when the standard deviation of split frequencies between the two simultaneous runs dropped below 0.01. Phylogenetic trees were visualized with FigTree v.1.4.0 (Rambaut 2012) and edited using Microsoft PowerPoint and Adobe Illustrator® CS6 v.26.0 (Adobe Systems, San Jose, CA, USA). The newly produced sequences were deposited in the GenBank nucleotide database (Table 1).

## ﻿Results

### ﻿Phylogenetic analysis

**Figure 1.**
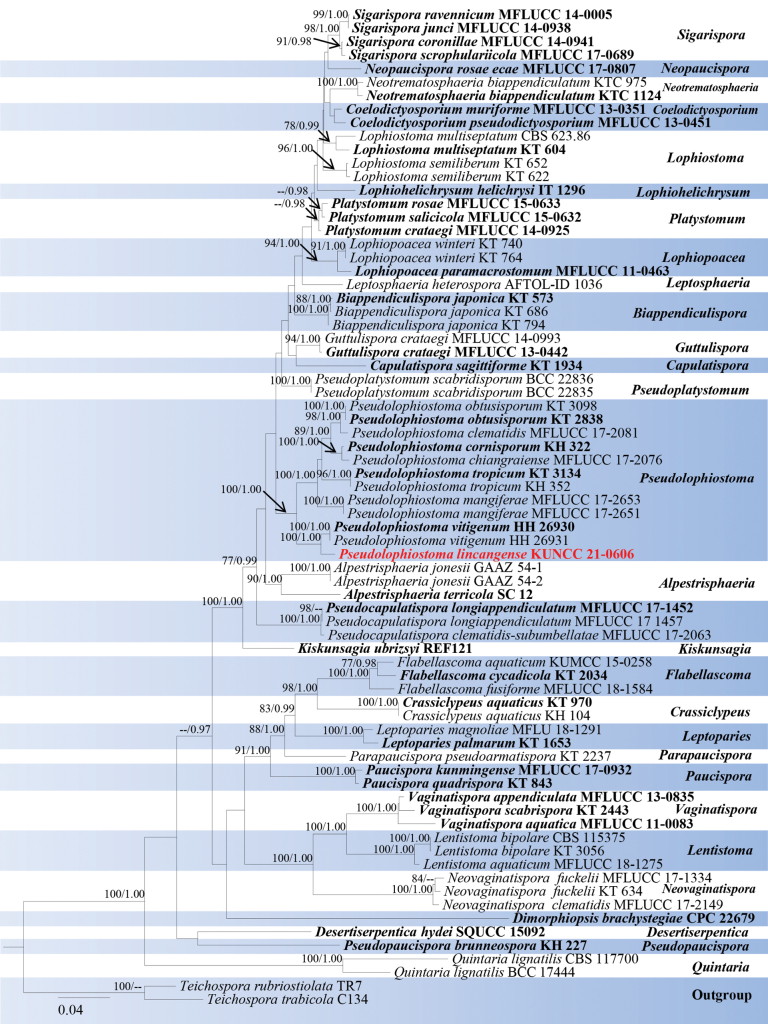
Phylogram generated from ML analysis based on SSU, LSU, ITS, *tef*1-α, and *rpb*2 sequence data representing the family Lophiostomataceae. Related sequences were obtained from Phukhamsakda et al. (2020). Bootstrap values for ML are equal to or greater than 75%, and posterior probability values are equal to or greater than 0.95 from the BYPP analysis labelled on the nodes. Strains of the newly described species are in red, while type strains are in bold. The tree is rooted with *Teichosporarubriostiolata* (TR7) and *T.trabicola* (C134).

Analyses 1, Lophiostomataceae phylogeny, was based on combined SSU, LSU, ITS, *tef*1-α, and *rpb*2. The final alignment contained 4,230 characters used for the phylogenetic analyses, including alignment gaps. The alignment contained 74 strains, and the tree was rooted with *Teichosporarubriostiolata* (TR7) and *T.trabicola* (C134). The RAxML analysis of the combined dataset yielded a best-scoring tree with a final ML optimization likelihood value of -29697.884081. The matrix had 1,584 distinct alignment patterns with 26.07% undetermined characters or gaps. The estimated base frequencies were as follows; A = 0.249681, C = 0.245034, G = 0.268293, T = 0.236992; substitution rates AC = 1.509197, AG = 3.725453, AT = 1.272039, CG = 1.297966, CT = 7.905766, GT = 1.0; gamma distribution shape parameter *α* = 0.192719 and tree-length = 0.192719. The tree topologies of combined sequence data obtained from ML and BI analyses were not significantly different. Our isolate, *Pseudolophiostomalincangense* (KUNCC 21-0606), was closer and sister to *P.vitigenum* strains (HH 26930, HH 26931) but formed a separate lineage with 100% ML bootstrap and 1.00 BYPP support (Fig. 1).

**Figure 2.**
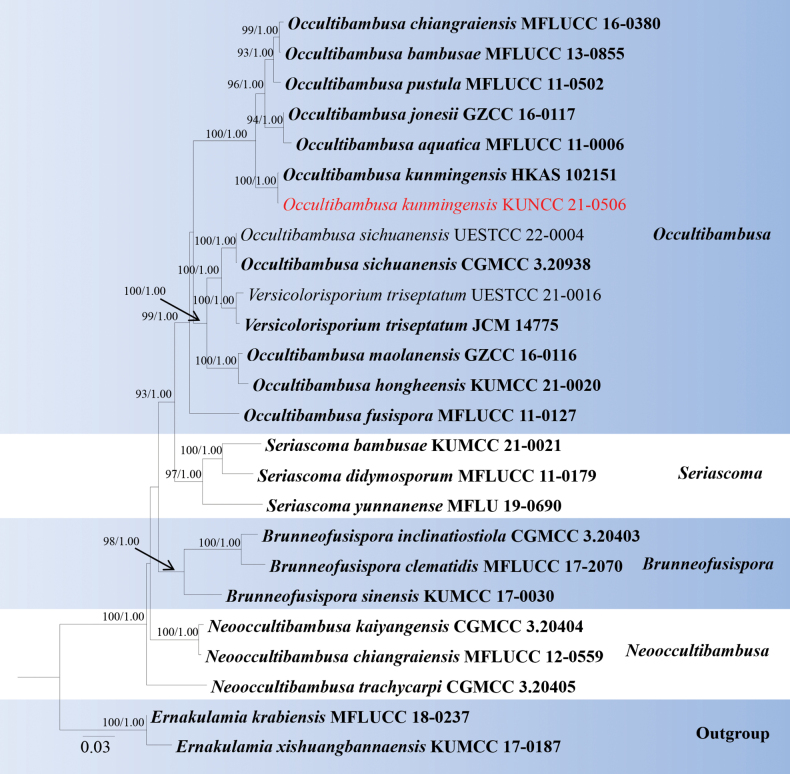
Phylogram generated from maximum likelihood analysis based on the combined SSU, LSU, ITS, *tef*1-α, and *rpb*2 dataset of Occultibambusaceae species. Related sequences were obtained from Yu et al. (2022). Bootstrap values for ML are equal to or greater than 75%, and posterior probability values are equal to or greater than 0.95 from the BYPP analysis labelled on the nodes. Strains of the newly described species are in red, while type strains are in bold. The tree is rooted with *Ernakulamiakrabiensis* (MFLUCC 18-0237) and *E.xishuangbannaensis* (KUMCC 17-0187).

Analyses 2, Occultibambusaceae phylogeny, was based on combined SSU, LSU, ITS, *tef*1-α, and *rpb*2. The final alignment contained 4,103 characters used for the phylogenetic analyses, including alignment gaps. The alignment contained 25 strains, and the tree was rooted with *Ernakulamiakrabiensis* (MFLUCC 18-0237) and *E.xishuangbannaensis* (KUMCC 17-0187). The RAxML analysis of the combined dataset yielded a best-scoring tree with a final ML optimization likelihood value of -16329.806421. The matrix had 1,047 distinct alignment patterns with 24.1% undetermined characters or gaps. The estimated base frequencies were as follows; A = 0.243761, C = 0.253253, G = 0.274093, T = 0.228893; substitution rates AC = 2.182200, AG = 2.182200, AT = 1.737406, CG = 1.445194, CT = 9.965966, GT = 1.0; gamma distribution shape parameter *α* = 0.155054 and tree-length = 0.951813. The tree topologies of combined sequence data obtained from ML and BI analyses were not significantly different. Our new isolate (KUNCC 21-0506) was nested with *Occultibambusakunmingensis* (HKAS 102151, type) and with 100% ML bootstrap and 1.00 BYPP support (Fig. 2).

**Figure 3.**
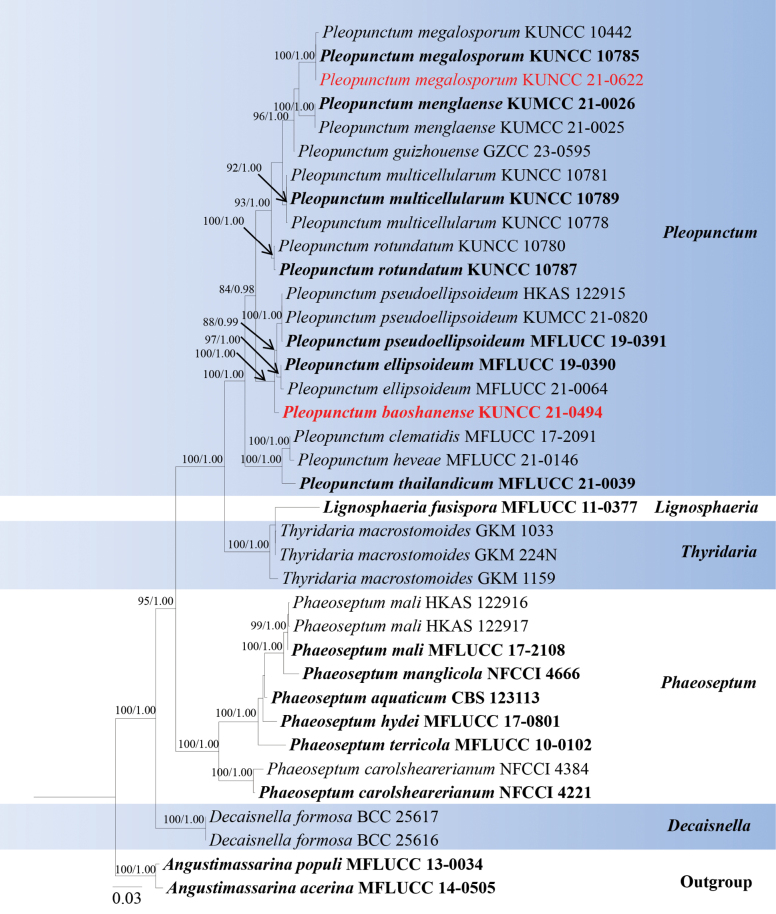
Phylogram generated from maximum likelihood analysis based on the combined SSU, LSU, ITS, *tef*1-α, and *rpb*2 dataset of Phaeoseptaceae species. Related sequences were obtained from Xu et al. (2023). Bootstrap values for ML are equal to or greater than 75%, and posterior probability values are equal to or greater than 0.95 from the BYPP analysis labelled on the nodes. Strains of the newly described species are in red, while type strains are in bold. The tree is rooted with *Angustimassarinaacerina* (MFLUCC 14-0505) and *A.populi* (MFLUCC 13-0034).

Analyses 3, Phaeoseptaceae phylogeny, was based on combined SSU, LSU, ITS, *tef*1-α, and *rpb*2. The final alignment contained 3,195 characters used for the phylogenetic analyses, including alignment gaps. The alignment contained 37 strains, and the tree was rooted with *Angustimassarinaacerina* (MFLUCC 14-0505) and *A.populi* (MFLUCC 13-0034). The RaxML analysis of the combined dataset yielded a best-scoring tree with a final ML optimization likelihood value of -12325.441217. The matrix had 882 distinct alignment patterns with 30.9% undetermined characters or gaps. The estimated base frequencies were as follows: A = 0.249369, C = 0.247445, G = 0.266516, T = 0.236670; substitution rates AC = 1.406931, AG = 4.076513, AT = 1.212607, CG = 1.303216, CT = 8.242374, GT = 1.000000; gamma distribution shape parameter *α* = 0.201687 and tree-length = 3.128705. The tree topologies of combined sequence data obtained from ML and BI analyses were not significantly different. Our isolate, *Pleopunctumbaoshanense* (KUNCC 21-0494), constituted a strongly supported (100% ML and 1.00 BYPP) independent lineage basal to *P.pseudoellipsoideum* (MFLUCC 19-0391, KUMCC 21-0820, HKAS122915) and *P.ellipsoideum* (97% ML and 1.00 BYPP). While our other isolate (KUNCC 21-0622) was grouped together with *P.menglaense* strains (KUNCC 1442, KUNCC 10785) with 100% ML bootstrap and 1.00 BYPP support (Fig. 3).

**Figure 4.**
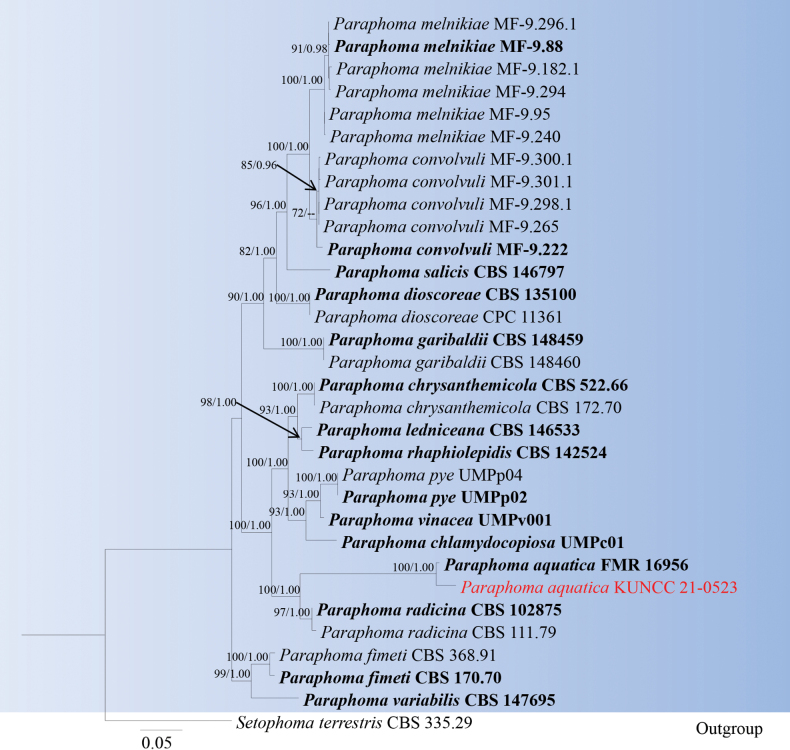
Phylogram generated from maximum likelihood analysis based on the combined LSU, ITS, *tef*1-α, *rpb*2 and *tub*2 dataset of *Paraphoma* species. Related sequences were obtained from previous publications (Magaña-Dueñas et al. 2021, Guarnaccia et al. 2022, and Gomzhina et al. 2020). Bootstrap values for ML are equal to or greater than 75%, and posterior probability values are equal to or greater than 0.95 from the BYPP analysis labelled on the nodes. Strains of the newly described species are in red, while type strains are in bold. The tree is rooted with *Setophomaterrestris* (CBS 335.29).

Analyses 4, *Paraphoma* phylogeny, was based on combined LSU, ITS, *tef*1-α, *rpb*2, and *tub*2. The final alignment contained 3,351 characters used for the phylogenetic analyses, including alignment gaps. The alignment contained 32 strains, and the tree was rooted with *Setophomaterrestris* (CBS 335.29). The RAxML analysis of the combined dataset yielded a best-scoring tree with a final ML optimization likelihood value of -13609.051798. The matrix had 929 distinct alignment patterns with 37.21% undetermined characters or gaps. The estimated base frequencies were as follows; A = 0.238385, C = 0.250275, G = 0.266033, T = 0.245306; substitution rates AC = 1.198498, AG = 2.769971, AT = 1.326456, CG = 0.861230, CT = 5.288549, GT = 1.0; gamma distribution shape parameter *α* = 0.274993 and tree-length = 1.180712. The tree topologies of combined sequence data obtained from ML and BI analyses were not significantly different. Our isolate, *Paraphomabaoshanenses* (KUNCC 21-0523), forms a distinct branch close to *P.aquatica* (FMR 16956) and with 100% ML bootstrap and 1.00 BYPP support (Fig. 4).

### ﻿Taxonomy

The present study introduces two new species and three new records. These taxa belong to the order Pleosporales and are described below.

#### ﻿ Pseudolophiostoma lincangense

Taxon classificationFungiPleosporalesLophiostomataceae

G.C. Ren & Tibpromma sp. nov.

D9F25B40-C2D3-57C0-9DF5-D09FA7A3E16E

Index Fungorum: IF902122

Facesoffungi Number: FoF15842

Fig. 5

##### Etymology.

The epithet refers to the location where the fungus was collected.

##### Holotype.

HKAS 122880.

##### Description.

***Saprobic*** on dead twigs of *Castanopsiscalathiformis* (Fagaceae) in terrestrial habitat. **Sexual morph: *Ascomata*** 280–330 µm high, 230–290 μm diam. (x̄ = 310 × 260 μm, n = 5), solitary or gregarious, immersed, papilla erumpent through host surface, subglobose, single or two locular, coriaceous, brown to dark brown, ostiolate. ***Ostioles*** 110–160 µm long, 80–90 μm diam., carbonaceous, mostly central, with crest-like opening, filled with hyaline periphyses. ***Peridium*** 10–20 µm wide, comprising 4–9 layers, composed of dark brown outer layers, inner layers comprising hyaline, flattened, angular, thick-walled cells of ***textura angularis***. ***Hamathecium*** composed of numerous, 1–2 µm wide, flamentous, septate, branched, cellular pseudoparaphyses. ***Asci*** 80–100 × 15–20 µm (x̄ = 94 × 16 µm, n = 20), 8-spored, bitunicate, fissitunicate, cylindrical, with pedicel, apically rounded, with a minute ocular chamber. ***Ascospores*** 32–40 × 5–5.8 µm (x̄ = 35 × 5.3 µm, n = 30), 1–2-seriate, fusiform, hyaline, straight or slightly curved, 1-septate, becoming 3-septate when germinated, constricted at the septa, narrower towards both end cells, upper cell longer than lower cell, guttulate, smooth-walled, with mucilaginous sheath. **Asexual morph**: Undetermined.

**Figure 5.**
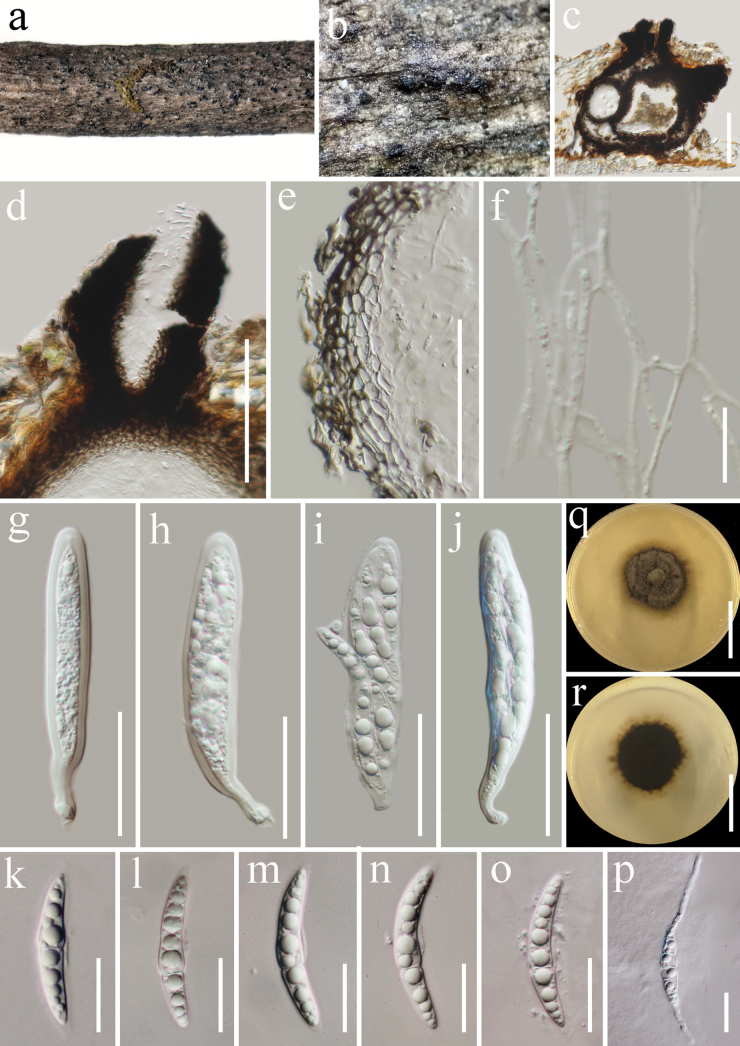
*Pseudolophiostomalincangense* (HKAS 122880, holotype) **a, b** appearance of ascomata on the host surface **c** section of ascoma **d** ostiole **e** peridium **f** pseudoparaphyses **g**–**j** asci **k**–**o** ascospores **p** germinated ascospore **q, r** culture characters on PDA (**q** = from above, **r** = from below). Scale bars: 100 μm (**c, d**); 50 μm (**e**); 10 μm (**f**); 30 μm (**g**–**j**); 15 μm (**k**–**p**); 30 mm (**q, r**).

##### Culture characteristics.

Ascospores germinated on PDA within 24 h at room temperature (25 °C). Germ tubes produced from the apical or the second cell of ascospore. ***Colonies*** on PDA, reaching 25 mm diameter after two weeks at 20–25 °C, mycelia superficial, circular, flat, fimbriate, undulate edge, gray with white gray at the center; reverse, dark green, pale yellow at the center.

##### Material examined.

China, Yunnan Province, Lincang (24°5'30"N, 100°5'33"E, elevation: 1557.49 m) on dead woody twigs of *Castanopsiscalathiformis* (Fagaceae), 12 July 2020, G.C. Ren, LC25 (HKAS 122880, holotype), ex-type living culture KUNCC 21-0606.

##### Notes.

Multi-loci phylogenetic analyses based on a concatenated SSU, LSU, ITS, *tef*1-α, and *rpb*2 sequence dataset show that our new collection (KUNCC 21-0606) clusters sister to strains of *Pseudolophiostomavitigenum* (HH 26930 and HH 26931) with 100% ML and 1.00 BYPP support (Fig. 1). Sequence comparison for the ITS region between *Pseudolophiostomalincangense* (KUNCC 21-0606) and *P.vitigenum* (HH 26930, type) showed a 2.67% (14/524 bp) base pair difference, 0.24% (2/848 bp) base pair difference for LSU region, 1.97% (17/863 bp) base pair difference for *tef*1-α region, but we were unable to compare *rpb*2 gene of *P.vitigenum* as there was no sequence data. Comparatively, the morphological characterization of *Pseudolophiostomalincangense* is similar to *P.vitigenum* in having immersed ascomata with papilla; carbonaceous ostiole with crest-like opening, and filled with hyaline periphyses; cylindrical asci with a short truncate pedicel and a minute ocular chamber; fusiform, hyaline, constricted at the septa, 1-septate ascospores (Thambugala et al. 2015). However, our new collection differs from *P.vitigenum* in having single or two locular ascomata, peridium consists of two layer cells of ***textura angularis***, smaller asci (94 × 16 μm vs. 129.8 × 20.1 μm) and ascospores (35 × 5.3 μm vs. 38.5 × 10.5 μm), and of the upper cell of the ascospores is longer than the lower cell (Thambugala et al. 2015). Therefore, we identify our collection as a new species from Yunnan Province, China.

#### ﻿ Occultibambusa kunmingensis

Taxon classificationFungiPleosporalesOccultibambusaceae

C.X. Liu, H. Zhang & K.D. Hyde, Fungal Diversity 105: 471.

4C3ABFCA-D6F9-5EC7-89FE-809A2AC9CCE2

Index Fungorum: IF557930

Facesoffungi Number: FoF09272

Fig. 6

##### Description.

***Saprobic*** on dead twigs of *Castanopsisdelavayi* (Fagaceae) in terrestrial habitat. **Sexual morph: *Ascomata*** 160–180 μm high, 160–240 μm diam. (x̄ = 175 × 195 μm, n = 5), dark brown to black, solitary or gregarious, semi-immersed to superficial, coriaceous, subglobose, with a short papillate, ostiolate. ***Ostioles*** 50–65 µm long, 35–40 μm diam., black, short. ***Peridium*** 20–35 μm thick, thin at the base and becoming wider laterally, composed of several layers of brown to dark brown, thick-walled cells of ***textura angularis***. ***Hamathecium*** 2.5–3.5 µm wide, hyphae-like, septate, cellular pseudoparaphyses, embedded in a gelatinous matrix. ***Asci*** (60)74–107(–116) × 12–14 μm (x̄ = 82 × 13 μm, n = 20), 8-spored, bitunicate, fissitunicate, clavate or cylindric-clavate, narrowly rounded at the apex, with a short truncate pedicel, apically rounded, with a minute ocular chamber. ***Ascospores*** 30–40 × 4–6 μm (x̄ = 35.6 × 5.3 μm, n = 20), overlapping 1–2-seriate, fusiform, straight or slightly curved, 1-septate, brown, constricted at the septa, guttulate, thin and smooth-walled, without mucilaginous sheaths and appendages. **Asexual morph**: Undetermined.

**Figure 6.**
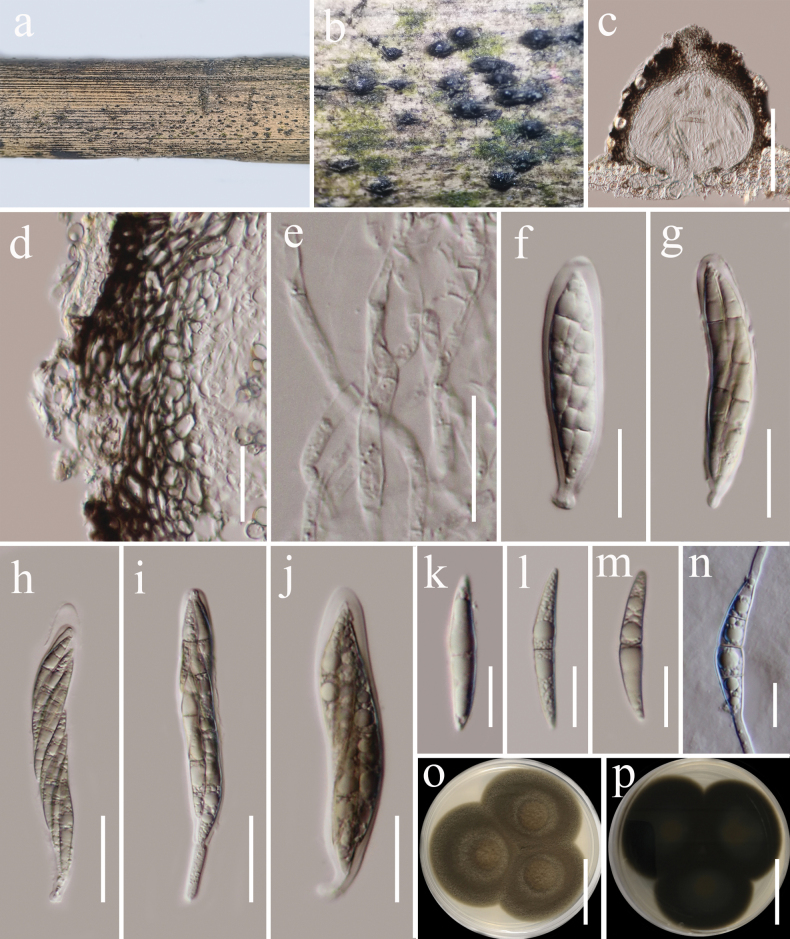
*Occultibambusakunmingensis* (HKAS 122706) **a** host **b** ascomata on host surface **c** vertical section of ascoma **d** peridium **e** pseudoparaphyses **f–j** asci **k–m** ascospores **n** germinated ascospore **o, p** colonies on PDA (**o** = from above, **p** = from below). Scale bars: 100 µm (**c**); 20 µm (**d–j**); 10 µm (**k–n**); 20 mm (**o, p**).

##### Culture characteristics.

Ascospores germinated on PDA within 24 h at room temperature (25 °C). Germ tubes produced from the basal and apical cells of ascospore. ***Colonies*** on PDA, reaching 25 mm diameter after 2 weeks at 20–25 °C, mycelia superficial, dense, circular, raised, entire edge, velvety, flossy; reverse black.

##### Material examined.

China, Yunnan Province, Baoshan (25°18'48"N, 99°09'50"E) on dead woody twigs of *Castanopsisdelavayi* (Fagaceae), 12 July 2020, G.C. Ren, BS17 (HKAS 122706), living culture KUNCC 21-0506.

##### Known host, habitats, and distribution.

Bamboo and *Castanopsisdelavayi* from freshwater and terrestrial in China (Dong et al. 2020; Jiang et al. 2021; this study).

##### Notes.

According to the multi-gene phylogenetic analyses of combined SSU, LSU, ITS, *tef*1-α, and *rpb*2 sequence dataset, our new isolate (KUNCC 21-0506) nested with *Occultibambusakunmingensis* (HKAS 102151), which was isolated from decaying bamboo submerged in freshwater in China (Dong et al. 2020) with 100% ML and 1.00 BYPP bootstrap support (Fig. 2). Our new isolate fits well with the morphological characteristics of the holotype of *O.kunmingensis* in having semi-immersed to superficial ascomata with short papillate, clavate or cylindric-clavate asci, fusiform, 1-septate, brown ascospores without mucilaginous sheaths and appendages (Dong et al. 2020). Sequence comparison for the ITS and *tef*1-α region between our isolates (KUNCC 21-0506) and *O.kunmingensis* (HKAS 102151) showed no significant base pair differences. Therefore, we identified our taxon as a new host record of *O.kunmingensis* from *Castanopsisdelavayi* (Fagaceae) in China, and it is the first record from woody litter.

#### ﻿ Pleopunctum baoshanense

Taxon classificationFungiPleosporalesPhaeoseptaceae

G.C. Ren & Tibpromma sp. nov.

9C3E4EA1-4C18-5462-9705-2C350BD58FB7

Index Fungorum: IF902123

Facesofungi Number: FoF15843

Fig. 7

##### Etymology.

The specific epithet “*baoshanense*” reflects the location “Baoshan” where the holotype was collected.

##### Holotype.

HKAS 134936.

##### Description.

***Saprobic*** on decaying wood of *Castanopsiscalathiformis* (Fagaceae) in terrestrial habitat. **Sexual morph**: Undetermined. **Asexual morph**: Hyphomycetous. ***Colonies*** on natural substrate sporodochial, superficial, black, scattered, gregarious. ***Mycelium*** immersed in the substratum, composed of septate hyphae. ***Conidiophores*** 3–5 µm wide (x̄ = 3.8 µm, n = 15), macronematous, mononematous, cylindrical, unbranched, septate, hyaline and smooth-walled. ***Conidiogenous cells*** monoblastic, terminal, integrated, hyaline. ***Conidia*** 33–46 × 22.5–27.6 μm (x̄ = 39 × 25 μm, n = 40), acrogenous, solitary, muriform, oval to ellipsoidal, smooth-walled, broadly obtuse at apex and dark brown, truncate at base and paler brown, often with a hyaline, elliptical to globose basal cell, 10–14 × 13–15 μm (x̄ = 12 × 13 μm, n = 30).

**Figure 7.**
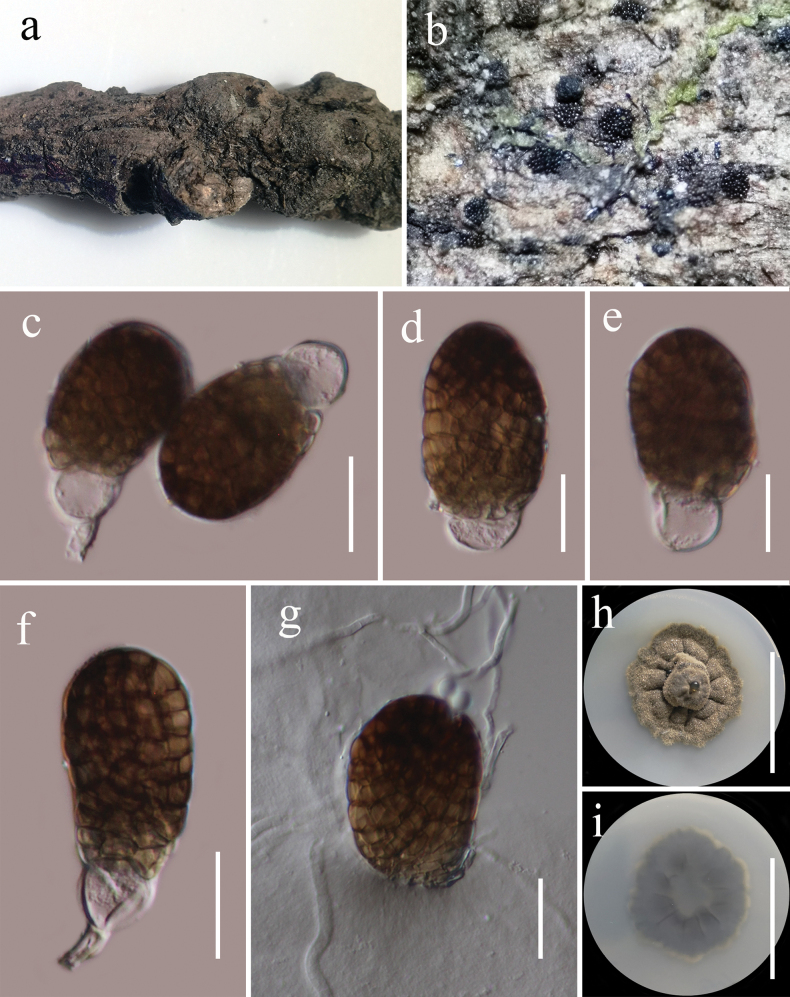
*Pleopunctumbaoshanense* (HKAS 134936, holotype) **a** host **b** colonies on the host surface **c–f** conidia with basal hyaline cells **g** germinated conidium **h, i** colony on PDA (**h** = from above, **i** = from below). Scale bars: 20 μm (**c–g**); 20 mm (**h, i**).

##### Culture characteristics.

Conidia germinated on PDA within 24 h at room temperature (25 °C). Germ tubes produced from the basal cells of conidia. ***Colonies*** on PDA, reaching 30 mm diameter after 2 weeks at 20–25 °C, mycelia superficial, irregular, umbonate at the center, fimbriate, undulate entire, grey, radially furrowed, with hyaline, glistening, rough, wrinkled, granular droplets of oil; reverse, grey, radially furrowed, grey white at the margin.

##### Material examined.

China, Yunnan Province, Baoshan (25°18'48"N, 99°09'50"E), on dead woody twigs of *Castanopsiscalathiformis* (Fagaceae), 12 July 2020, G.C. Ren, B194 (HKAS 134936, holotype), ex-type living culture KUNCC 21-0494.

##### Notes.

Multi-loci phylogenetic analyses based on a concatenated SSU, LSU, ITS, *tef*1-α, and *rpb*2 sequence dataset show that our new collection (KUNCC 21-0494) clusters sister to strains of *Pleopunctumpseudoellipsoideum* (MFLUCC 19-0391, KUMCC 21-0820, HKAS 122915) and *P.ellipsoideum* (MFLUCC 19-0390, MFLUCC 21-0064) with solid support (100% ML and 1.00 BYPP, Fig. 3). Sequence comparison for the ITS region between *Pleopunctumbaoshanense* (KUMCC 21-0494) and *P.pseudoellipsoideum* (MFLUCC 19-0391) showed a 1.54% (8/520 bp, without the gaps) base pair difference, 2.26% (21/930 bp, without the gaps) base pair difference for the *tef*1-α region. Sequence comparison for the ITS region between *Pleopunctumbaoshanense* (KUNCC 21-0494) and *P.ellipsoideum* (MFLUCC 19-0390) showed a 1.65% (8/486 bp, without the gaps) base pair difference, 1.81% (15/827 bp, without the gaps) base pair difference for *tef*1-α region. *Pleopunctumbaoshanense*, *P.pseudoellipticum*, and *P.ellipsoideum* are morphologically similar, they have sporodochial conidiomata; septate hyphae mycelium; mononematous, cylindrical conidiophores; monoblastic, terminal, hyaline conidiogenous cells and muriform, oval to ellipsoidal conidia often with a hyaline, elliptical to globose basal cell, but can be distinguished from *P.pseudoellipsoideum* and *P.ellipsoideum* in having hyaline conidiophores and conidiogenous cells, different sizes of conidia (39 × 25 μm vs. 50 × 24 μm vs. 45 × 20 μm), while both of them have medium brown conidiophores and conidiogenous cells (Liu et al. 2019; Xu et al. 2023). Therefore, based on morphology and phylogenetic distinctiveness, we introduce *Pleopunctumbaoshanense* as a new species.

#### ﻿ Pleopunctum megalosporum

Taxon classificationFungiPleosporalesPhaeoseptaceae

R.J. Xu, Q. Zhao & Boonmee Journal of Fungi 9 (5, no. 560): 9 (2023)

98D6AF85-D36C-514A-8C17-BDC3149E1A0F

Index Fungorum: IF847826

Facesofungi Number: FoF14063

Fig. 8

##### Description.

***Saprobic*** on dead twigs of *Castanopsiscalathiformis* (Fagaceae) in terrestrial habitat. **Sexual morph**: Undetermined. **Asexual morph**: Hyphomycetous. ***Colonies*** on host, sporodochial, superficial, light brown, scattered, and punctiform. ***Mycelium*** immersed in the substratum, composed of septate, branched, sub hyaline to light brown hyphae. ***Conidiophores*** 13–66 × 2.6–4.2 µm (x̄ = 37.7 × 3.5, n = 20), macronematous, mononematous, hyaline to light brown, cylindrical, unbranched, septate, smooth-walled. ***Conidiogenous cells*** 3.9–5.5 × 3.6–4.7 µm (x̄ = 4.6 × 4 µm, n = 20), monoblastic, terminal, light brown. The conidia are dimorphic, acrogenous, and solitary. ***α conidia*** 30–40 × 10–15 µm (x̄ = 33 × 12.5 µm, n = 30), hyaline to light brown, muriform, oblong to obovate, constricted at septa, slightly obtuse to rounded at apex. ***β conidia*** 48–60 × 24–31 µm (x̄ = 54.7 × 27.3 µm, n = 30), brown, muriform, oval to long elliptical, slightly constricted at septa, often with a hyaline, elliptical to globose, 0–multiple-basal cells, 12.5–17.6 × 7.7–11 µm (x̄ = 14.7 × 9.2 µm, n = 20).

**Figure 8.**
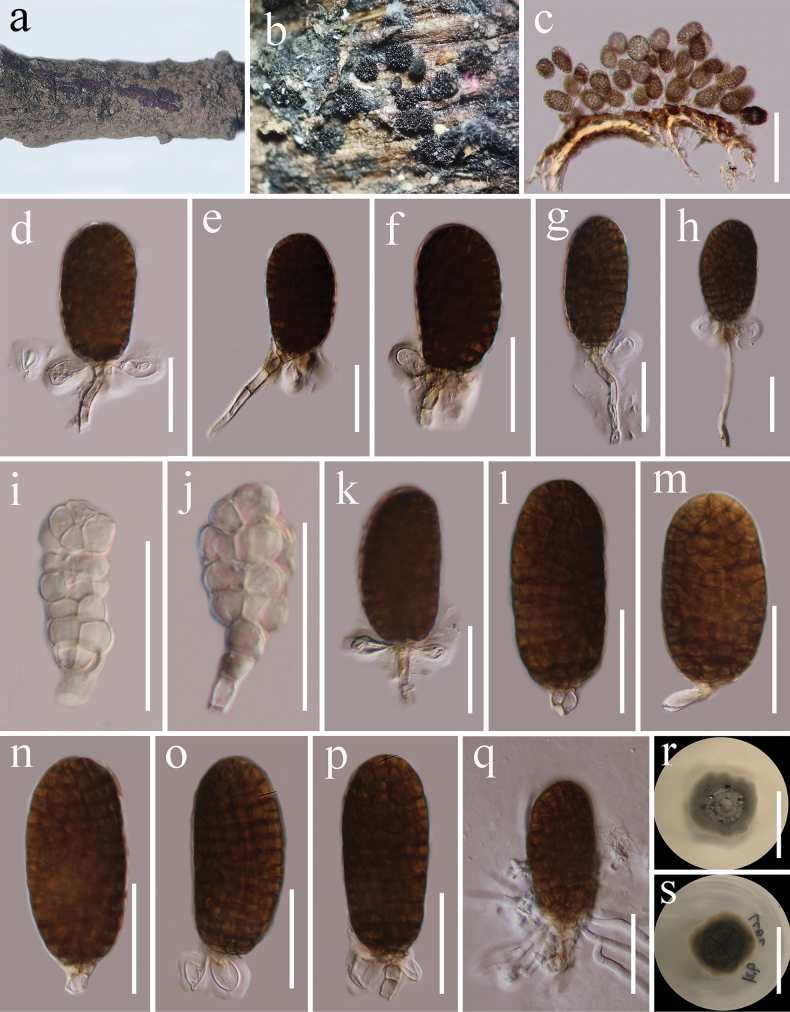
*Pleopunctummegalosporum* (HKAS 134935) **a** host **b** colonies on the host surface **c** conidia on substrate **d–h, k–p** β conidia with basal cells and conidiophores **i, j** α conidia showing remnant of conidiogenous cells at base **q** germinated conidium **r, s** colony on PDA (**r** = from above, **s** = from below). Scale bars: 100 μm (**c**); 30 μm (**d–q**); 20 mm (**s, r**).

##### Culture characteristics.

Conidia germinated on PDA within 24 h at room temperature (25 °C). Germ tubes produced from the basal cells of conidia. ***Colonies*** on PDA, reaching 30 mm diameter after 2 weeks at 20–25 °C, mycelia superficial, irregular, slightly umbonate at the center, fimbriate, undulate edge, grey at the margin, grey white at the center, with hyaline, glistening, granular droplets of oil; reverse, atrovirens, notably radially furrowed, golden brown at the margin.

##### Material examined.

China, Yunnan Province, Lincang (24°5'30"N, 100°5'33"E, elevation: 1557.49 m), on dead woody twigs of *Castanopsiscalathiformis* (Fagaceae), 12 July 2020, G.C. Ren, LC62 (HKAS 134935), living culture KUNCC 21-0622.

##### Known host, habitats, and distribution.

*Cryptocaryaacutifolia*, freshwater and terrestrial, China (Xu et al. 2023; this study).

##### Notes.

*Pleopunctummegalosporum* was introduced by Xu et al. (2023) from submerged decaying wood in a freshwater stream in China. Our collection (KUMCC 21-0622) resembles *P.megalosporum* (KUMCC22-10799) in having sporodochial conidiomata; septate, subhyaline to light brown mycelium; mononematous, cylindrical, light brown conidiophores; monoblastic, terminal, light brown conidiogenous cells and muriform, oval to long elliptical conidia often with a hyaline, elliptical to globose, 0–multiple-basal cell (Xu et al. 2023). Multi-loci phylogenetic analyses based on a concatenated SSU, LSU, ITS, *tef*1-α, and *rpb*2 sequence dataset show that our new collection (KUNCC 21-0622) clusters with *Pleopunctummegalosporum* (KUNCC 10785, KUNCC 10442) with strong support (100% ML and 1.00 BYPP, Fig. 3). Sequence comparison for the ITS and *tef*1-α region between our isolate (KUNCC 21-0622) and *Pleopunctummegalosporum* (KUNCC 10785) showed no significant base pair differences. Therefore, we introduce our collection as the first record of *P.megalosporum* from *Castanopsiscalathiformis* (Fagaceae) in China.

#### ﻿ Paraphoma aquatica

Taxon classificationFungiPleosporalesPhaeosphaeriaceae

Magaña-Dueñas, Stchigel & Cano-Lira Journal of Fungi 7(12, no. 1102): 9 (2021)

CEB03285-7CA4-5B1C-B15D-01062F4A87E4

Index Fungorum number: IF841364

FacesofFungi number: FoF15844

Fig. 9

##### Description.

***Saprobic*** on dead woody twigs of *Castanopsisdelavayi* (Fagaceae). **Sexual morph: *Ascomata*** 110–130 × 120–130 μm (x̄ = 122 × 126 μm, *n* = 5), solitary, scattered, erumpent to immersed, uni-loculate, globose to subglobose, black. ***Ostioles*** central. ***Peridium*** 7–12 μm wide, thin, comprising 3–4 layers of light brown to brown cells of ***textura prismatica***. ***Hamathecium*** 1.5–2.5 μm wide, cylindrical, filiform, hyaline, septate, branched, cellular pseudoparaphyses, embedded in a gelatinous matrix. ***Asci*** 45–63 × 9–11 μm (x̄ = 55 × 9.5 μm, *n* = 20), 8-spored, bitunicate, fissitunicate, clavate, slightly broad at center, apically rounded, with short and rounded pedicel, minute ocular chamber. ***Ascospores*** 19–22 × 4–4.6 μm (x̄ = 20.7 × 4.2 μm, *n* = 30), overlapping, biseriate, hyaline, narrowly fusiform, straight or slightly curved, with 3 transverse septa, enlarged at the second cell, constricted at the septa, smooth-walled, guttulate, without a mucilaginous sheath. **Asexual morph**: see Magaña-Dueñas et al. (2021).

**Figure 9.**
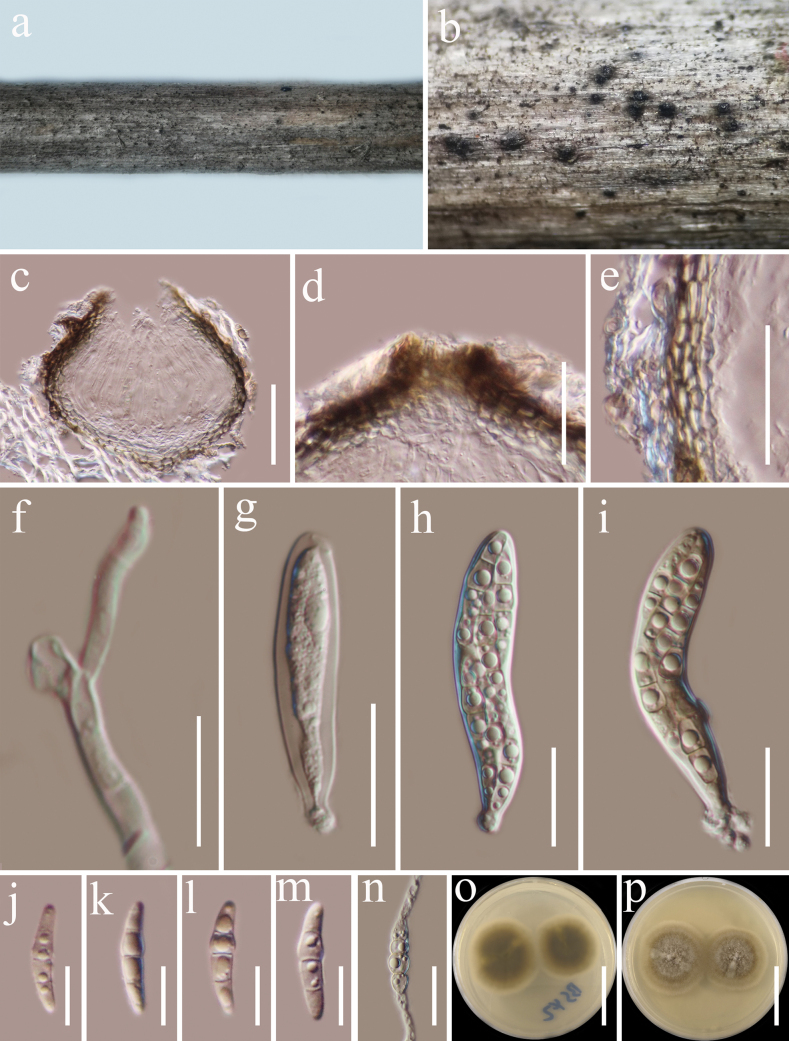
*Paraphomaaquatica* (HKAS 122713) **a** host **b** ascomata on the host surface **c** vertical section of ascoma **d** ostiole **e** peridium **f** pseudoparaphyses **g–i** asci **j–m** ascospores **n** germinated ascospore **o, p** culture characters on PDA (**o** = from above, **p** = from below). Scale bars: 50 µm (**c**); 30 µm (**d, e**); 10 µm (**f, j–n**); 20 µm (**g–i**); 20 mm (**o, p**).

##### Culture characteristics.

Ascospores germinated on PDA within 24 h at room temperature (25 °C). Germ tubes produced from the basal and apical cells of ascospore. ***Colonies*** on PDA, reaching 25 mm diameter after 2 weeks at 20–25 °C, mycelia superficial, medium density mycelia, entire margin, umbonate at center, band, rough surface, velvety, raised, grayish yellow, white mycelium attached to the central surface; reverse atrovirens.

##### Material examined.

China, Yunnan Province, Baoshan (25°18'48"N, 99°09'50"E), on dead woody twigs of *Castanopsisdelavayi* (Fagaceae), 12 July 2020, G.C. Ren, BS42 (HKAS 122713), living culture KUNCC 21-0523.

##### Known host, habitats, and distribution.

Capafonts and *Castanopsisdelavayi*, freshwater submerged plant debris and terrestrial, China and Spain (Magaña-Dueñas et al. 2021; this study).

##### Notes.

Multi-loci phylogenetic analyses based on a concatenated LSU, ITS, *tef*1-α, *rpb*2, and *tub*2 sequence dataset show that our new collection (KUNCC 21-0523) formed a sister lineage to the ex-type strain of *Paraphomaaquatica* (FMR 16956) with solid support (100% ML and 1.00 BYPP, Fig. 4). Sequence comparison for the ITS region between *Paraphomabaoshanenses* (KUNCC 21-0523) and *P.aquatica* (FMR 16956) showed a 6.17% (30/486 bp) base pair difference, 0.12% (2/848 bp) base pair difference for LSU region, 2.06% (9/437 bp) base pair difference for the *tef*1-α region. Unfortunately, the morphology could not be compared as *Paraphomaaquatica* was reported in its asexual morph with no information on its sexual morph (Magaña-Dueñas et al. 2021). The species of *Paraphoma* were introduced from its asexual morph (Morgan-Jones and White 1983; de Gruyter et al. 2010; Quaedvlieg et al. 2013; Moslemi et al. 2016, 2018; Crous et al. 2017, 2021a, 2021b; Gomzhina et al. 2020; Magaña-Dueñas et al. 2021; Guarnaccia et al. 2022), while we introduced our new collection from its sexual morph. Therefore, we could not compare our new collection with other *Paraphoma* species. However, based on the phylogenetic distinctiveness, we introduce our collection as the first record of *P.aquatica* from *Castanopsisdelavayi* (Fagaceae) in China, and our species is the first sexual morph recorded in this genus.

## ﻿Discussion

This study introduces two new species of woody litter fungi: *Pseudolophiostomalincangense*, and *Pleopunctumbaoshanense* from Yunnan Province, China. We also report new host records of *Occultibambusakunmingensis* on *Castanopsisdelavayi*, *Pleopunctummegalosporum* on *Castanopsiscalathiformis* for the first time in China, and *Paraphomaaquatica* is the first sexual morph of *Paraphoma* on *Castanopsisdelavayi*.

*Pseudolophiostoma* was introduced by Thambugala et al. (2015), with *P.vitigenum* designated as the type species. Currently, this genus comprises seven accepted species (Species Fungorum 2024). *Pseudolophiostoma* species are typically saprobes found on various herbaceous, woody, or vine plants such as Bidenspilosavar.radiata, *Clematisfulvicoma*, *Livistonaboninensis*, *Stachytarphetajamaicensis*, and *Vitiscoignetiae*, and are distributed across China (Taiwan), Japan, and Thailand (Thambugala et al. 2015; Hashimoto et al. 2018; Tennakoon et al 2018; Phukhamsakda et al. 2020). *Pseudolophiostoma* species are known only by their sexual morph, and their asexual morph has not been discovered yet (Thambugala et al. 2015). Therefore, the asexual morph of *Pseudolophiostoma* remains uncertain and thus, further studies with additional collections are needed to understand the asexual morph.

*Occultibambusa*, introduced by Dai et al. (2017) with *O.bambusae* as the type species, is commonly found on bamboo culms. These species have a wide distribution, particularly in China and Thailand, where they inhabit both freshwater and terrestrial habitats (Hyde et al. 2016; Dai et al. 2017; Zhang et al. 2017; Dong et al. 2020; Jiang et al. 2021; Yu et al. 2022). Currently, Species Fungorum (2024) recognizes 10 *Occultibambusa* species. Most of these species have been reported based on their sexual morphs, with only *O.fusispora* known from its holomorph, and the coelomycetous asexual morph discovered in culture (Dai et al. 2017; Jiang et al. 2021). In this study, our collection also reports sexual morph and thus, further studies are needed to understand the asexual morph with additional fresh collections.

*Pleopunctum* was introduced as the first hyphomycetous genus in Phaeoseptaceae by Liu et al. (2019) to include two species: *P.ellipsoideum* and *P.pseudoellipticum*. Subsequently, nine *Pleopunctum* species viz. *P.bauhiniae*, *P.clematidis*, *P.guizhouense*, *P.heveae*, *P.megalosporum*, *P.menglaense*, *P.multicellularum*, *P.rotundatum* and *P.thailandicum* were accepted (Phukhamsakda et al. 2020; Boonmee et al. 2021; Koukol and Delgado 2021; Senwanna et al. 2021; Wanasinghe et al. 2021; Xu et al. 2023; Zhang et al. 2023). Currently, eleven *Pleopunctum* species are accepted in Species Fungorum (2024) and all have been reported in their asexual morph (Liu et al. 2019). *Pleopunctum* species distributed in China and Thailand and saprobic on dead wood of *Bauhiniavariegata* (Fabaceae), *Clematissikkimensis* (Ranunculaceae), *Heveabrasiliensis* (Euphorbiaceae) and some unknown woody litter in freshwater and terrestrial habitats (Hyde et al. 2019; Phukhamsakda et al. 2020; Senwanna et al. 2021; Xu et al. 2023). *Pleopunctumbauhiniae*, *P.clematidis*, *P.heveae*, *P.megalosporum*, and *P.menglaense* have dimorphic conidia on the natural substrate (Hyde et al. 2019; Phukhamsakda et al. 2020; Senwanna et al. 2021; Wanasinghe et al. 2021; Xu et al. 2023). However, *P.ellipsoideum*, *P.guizhouense*, *P.multicellularum*, *P.pseudoellipticum*, *P.rotundatum* and *P.thailandicum* are characterized by one conidium type and share very similar morphological characteristics (Liu et al. 2019; Boonmee et al. 2021; Koukol and Delgado 2021; Xu et al. 2023). The phenotypic variation among strains, influenced by environmental factors, can make morphological differentiation challenging (Hyde et al. 2020). However, our research has shown that molecular sequence data are a reliable tool for identifying *Pleopunctum* species. This confidence-inspiring finding, supported by previous publications and our study (Boonmee et al. 2021; Koukol and Delgado 2021; Xu et al. 2023).

*Paraphoma* was established in 1983 with *P.radicina* as the type species (Morgan-Jones and White 1983). Subsequently, fourteen species are accepted in *Paraphoma*. Currently, fifteen *Paraphoma* species are accepted in Species Fungorum (2024). *Paraphoma* species are widely distributed worldwide, for instance in Australia, China, Czech Republic, Italy, the Netherlands, New Zealand, Russia, South Korea, Spain, Switzerland, Ukraine, the United Kingdom and the United States. These species include endophytes, pathogens, and saprobes on the plant of *Atractylodeslancea*, *Buxussempervirens*, *Chrysanthemummorifolium*, *Campanularapunculoides*, Convolvulaceae sp., *Dioscoreatokoro*, *Juniperuscommunis*, *Paraphomavinacea*, *Rhaphiolepsisindica*, *Strawberry*, Salixcf.alba, *Tanacetumcinerariifolium*, soil and dung (de Gruyter et al. 2010; Bensch et al. 2012; Quaedvlieg et al. 2013; Crous et al. 2017, 2021b; Moslemi et al. 2018; Gomzhina et al. 2020; Guarnaccia et al. 2022). Currently, only asexual morph has been reported from *Paraphoma* species (Quaedvlieg et al. 2013; Gomzhina et al. 2020). However, we have discovered the sexual morph of *Paraphomaaquatica*, which is saprobic on dead wood of *Castanopsiscalathiformis* (Fagaceae), and this is the first sexual morph recorded in *Paraphoma*.

## Supplementary Material

XML Treatment for Pseudolophiostoma lincangense

XML Treatment for Occultibambusa kunmingensis

XML Treatment for Pleopunctum baoshanense

XML Treatment for Pleopunctum megalosporum

XML Treatment for Paraphoma aquatica

## ﻿Additional information

### Author ORCIDs

Guang-Cong Ren https://orcid.org/0000-0001-9923-2626

Saowaluck Tibpromma https://orcid.org/0000-0002-4706-6547

Samantha C. Karunarathna https://orcid.org/0000-0001-7080-0781

Abdallah M. Elgorban https://orcid.org/0000-0003-3664-7853

### Data availability

All of the data that support the findings of this study are available in the main text.
